# The Blues and Older Minority Musicians XXVIII: More Than Just Music

**DOI:** 10.1093/geroni/igab046.2257

**Published:** 2021-12-17

## Abstract

The GSA “ ‘Bo Diddley’ Track” goes virtual again to present its 28th consecutive Annual Scientific Meeting of outstanding music, personal and musical narrative, and fun regardless of hurricanes, recessions, riots (ok-ok-so it was just a little good-natured audience rowdiness at one of the performances at GSA). Instead of the Rhythm Room or Warsaw Wally’s in Phoenix, the program returns to Chicago on 11/13, 6pm Eastern with the “Sweetheart of the Blues” - Shirley Johnson, one of the famous “Mojo Mamas” performing regularly at BLUE Chicago since the 1990s. Like so many Blues artists, she started in church at age 10 and later, influenced by Mahalia Jackson, Koko Taylor, Etta James, and Ruth Brown, her music is described as a “gritty, big-voiced blues singer” who also reaches heights of “soulful and smooth”. She turns everything she does into a work of art. Grab your favorite beverage and join us for rousing music and a great party. No rioters allowed!

